# Exploring the Scientific Interest for Olive Oil Origin: A Bibliometric Study from 1991 to 2018

**DOI:** 10.3390/foods9050556

**Published:** 2020-05-01

**Authors:** Astrid Maléchaux, Yveline Le Dréau, Jacques Artaud, Nathalie Dupuy

**Affiliations:** Aix Marseille Univ, Avignon Université, CNRS, IRD, IMBE, 13397 Marseille, France; yveline.le-dreau@univ-amu.fr (Y.L.D.); jacques.artaud@univ-amu.fr (J.A.); nathalie.dupuy@univ-amu.fr (N.D.)

**Keywords:** olive oil, bibliometrics, clustering, keywords analysis, network of authors

## Abstract

The authenticity and traceability of olive oils have been a growing concern over the past decades, generating numerous scientific studies. This article applies the tools of bibliometric analyses to explore the evolution and strategic orientation of the research focused on olive oil geographical and varietal origins. A corpus of 732 papers published in 178 different journals between 1991 and 2018 was considered. The most productive journals, authors and countries are highlighted, as well as the most cited articles associated with specific analytical techniques. A cluster analysis on the keywords generates 8 main themes of research, each focused on different analytical techniques or compounds of interest. A network between these thematic clusters and the main authors indicates their area of expertise. The metabolomics methods are drawing increasing interest and studies focused on the relationships between the origin and the sensory or nutritional properties provided by minor compounds of olive oils appear to be future lines of research.

## 1. Introduction

Olive oils and their composition in relation to their geographic or varietal origins have been extensively studied in recent years, as part of the food authenticity topic. Indeed, consumers have been paying increasing attention to the quality of the food products they buy. Marketing and sociology studies show that consumers rely on their sensory perceptions and on external information, like nutritional values or certifications present on the label, to assess food quality. Furthermore, the concepts of “quality”, “safety”, “traceability” and “authenticity” are not clearly defined by consumers and are often related with each other [1]. Authenticity is also associated with more “natural” and “healthy” products, for which consumers are willing to pay a higher price. Many aspects such as the production method, geographical origin or variety of the ingredients, play a role in the perception of food authenticity and can also be viewed as part of a cultural heritage, inciting to buy local or traditional food products [2]. Therefore, ensuring consistency between the label and the actual content of a food product is crucial in maintaining the consumers trust. In the case of olive oil, European regulation requires the geographical origin (EU member state or third country) and quality grade (extra virgin, virgin, blend of refined and virgin or pomace oil) to be specified on the label [3]. Moreover, additional information such as a protected designation of origin (PDO) or geographical indication (PGI) can be present. Authenticity and traceability are essential since the attribution of a PDO is based on specific rules regarding the geographical and varietal origins for each designation [4]. Thus, there is a wealth of scientific studies focusing on the determination of olive oil origin and some challenges in this area have been introduced in a previous literature review [5]. However, the evolution of this research topic should be further analyzed by means of bibliometric methods [6]. Indeed, as explained in previous bibliometric studies, the exploration of the structure of a research field can provide strategic insight on a subject [7,8]. The literature associated with the fields of olive oil and determination of origin is extremely important. Rapid screening methods using vibrational spectroscopy and/or specific methods based on chromatography can be used to detect adulterations and various compounds including triglycerides, fatty acids, phenolic compounds, vitamins, etc., can be analyzed. Different methodologies have been applied to perform these researches, and they have evolved over time. It is thus essential to perform a bibliometric analysis to understand the publishing and citation trends of this research field. The articles that associate olive oils with their varietal or geographical origins were identified and show that the research pathways chosen to treat the problem vary over time. Moreover, the most cited articles—as well as the authors who have contributed to a large number of papers—were highlighted. Finally, the main keywords were used to conduct a cluster analysis representing the network of relationships between the authors and their main themes of research. The aim of this study is to strategically position future research through the identification of declining and emerging thematic clusters.

## 2. Materials and Methods

References were obtained using the Web of Science database on 12 June 2019. The following terms were researched in the “Topic” field, which gathers the “Title”, “Abstract” and “Keywords” fields:

(“olive oil” OR “olive oils”) AND origin AND (cultivar OR variet* OR geograph*)

The timespan was limited to studies published between 1991 and 2018, since no relevant articles were found in the database before 1991. This query yielded 732 references, classified by Web of Science^®^ into 653 scientific articles, 51 reviews, 25 proceedings papers and 3 notes or corrections, all of which will be referred to as “articles” for this study. The full citation records were exported in order to be treated by the Matheo Analyzer^®^ software (Matheo Software, Marseille, France) [9]. This bibliometric study can provide strategic orientation for future research, although it should be considered with caution due the limits of the database used to retrieve the studied articles. The results from different databases could not be merged because of the differences in the methods used to collect citations, which were incompatible with the treatment by Matheo Analyzer^®^.

## 3. Results and Discussion

### 3.1. Evolution of Keywords

**Figure 1:**
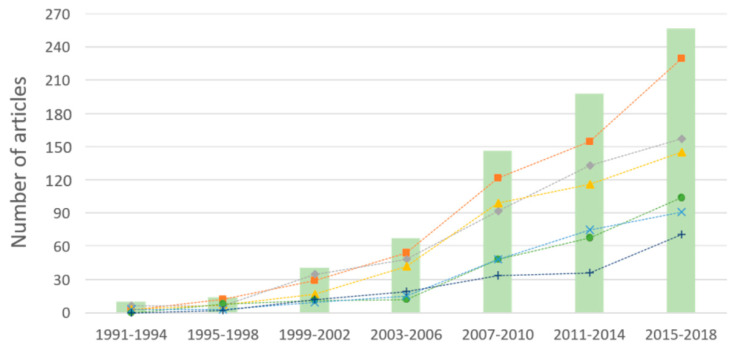
Evolution of the total number of articles (bars) and number of articles containing keywords related to specific subjects (■: analytical techniques, ♦: chemometrics, ▲: chemical compounds, ●: authenticity and quality, ×: geographical origin, +: varietal origin) between 1991 and 2018.

Figure 1 shows the number of articles studying the varietal or geographical origins of olive oils. It has been steadily increasing since the first publication by Alberghina et al. in 1991 [10]. This progress has been especially important since 2007, leading to the publication of more than 60 articles each year between 2015 and 2018. Figure 1 also transcribes the evolution of the number of articles during the studied period according to the types of keywords used. Articles were grouped together under generic terms. The number of articles grouped under the term “analytical techniques”, which encompasses articles using either spectral methods or chromatographic methods, has grown much more than the number of articles mentioning other keywords. Otherwise, a significant number of articles also mention the use of chemometrics modeling or target particular compounds of olive oils that could be markers of their origin. Some articles employ general keywords indicating their interest in olive oil authenticity, traceability or quality, while other publications specify whether they focus on geographical or varietal origins. In this case, a stronger increase of the number of articles dealing with geographical origin compared to varietal origin can be observed since 2007.

Table 1 focuses on the number of articles containing keywords related to specific analytical techniques. These categories were obtained after a time-consuming manual grouping of many keywords, most of which only appeared in one article, showing that spelling disparities and the choice of very specific keywords can be an issue for bibliometric studies. Moreover, 67 articles did not have any “keywords” section, either because of an oversight or because no keywords were required by their format of publication, and they were thus not taken into account.

Nuclear magnetic resonance spectroscopy was the most popular analysis, mentioned in 75 articles. There appears to be a strong interest in sensory analysis with 57 articles, but olfactometric measurements were almost as often conducted with electronic sensors (e-nose and e-tongue, found in 41 articles). DNA analysis was also at the center of many studies, with 49 articles containing keywords related to this subject. Gas and liquid chromatographies were sensibly as popular, with 34 and 31 articles, respectively, and often associated with detection by mass spectrometry. Another important application of mass spectrometry was for isotope ratio analysis, with 40 articles. Regarding vibrational spectroscopic techniques, mid- and near-infrared were often used, with 40 and 28 articles, respectively. Finally, there was a marginal interest for UV-Visible, Raman and fluorescence spectroscopies, each appearing in less than 20 articles. The most cited articles for each technique can be retrieved (Table 1) [11,12,13,14,15,16,17,18,19,20,21,22]. Their publication dates range from 1997 for NMR [11] to 2011 for fluorescence spectroscopy [22] and the number of citations by the end of 2018 were between 80 for UV-Visible spectroscopy [20] and 301 for electronic sensors [14].

### 3.2. Core Journals

The 732 articles of the corpus were published in 178 different journals between 1991 and 2018, with 57% of the journals having only one article dealing with olive oil varietal or geographical origins. The 15 journals having published at least 10 articles on the subject were presented in Table 2. The impact factors (IF) and subject category quartile ranks (Q, indicating the rank of the considered journal compared to the other journals in its subject category) reported therein were retrieved from InCites Journal Citation Reports (Clarivariate Analytics). Among these main journals, Food Chemistry and the Journal of Agricultural and Food Chemistry lead the rankings, with 94 and 66 articles, respectively. The journal with the highest IF as of 2018 was Critical Reviews in Food Science and Nutrition, with an IF over 6, followed by Food Chemistry and Analytica Chimica Acta, with IF over 5. Grasas y Aceites and Rivista Italiana delle Sostanze Grasse were the only journals having published more than 10 articles with an IF under 1, indicating the strong scientific interest for olive oil authenticity their respective countries of origin (Spain and Italy). The subject categories assigned in the Journal Citation Reports indicate the general research area of each journal. Some journals focus only on one area, while others were multidisciplinary since they were included in several subject categories. The most common category appears to be “food science”, with 12 of the 15 journals concerned. Other subjects of interest include “applied chemistry”, “analytical chemistry”, “nutrition”, “agriculture” and “biochemical research methods”. The most cited articles from each journal were also presented in Table 2 [12,14,23,24,25,26,27,28,29,30,31,32,33,34,35]. The citation count by the end of 2018 indicates that journals with the highest IF do not necessarily have highest number of citations. Indeed, the most cited article was a kinetic study of the radical scavenger capacity of different oils published in the Journal of Agricultural and Food Chemistry [24] with and IF of 3.571 and 405 citations by the end of 2018, followed by a review on electronic sensors in Analytica Chimica Acta [14] with an IF of 5.256 and 301 citations by the end of 2018. Among the most cited articles, the oldest one was published in 1993 in the Journal of the Science of Food and Agriculture and deals with the classification of geographical origin based on fatty acid profiles obtained by gas chromatography [28]. The two most recent articles were published in 2014 in the Journal of Oleo Science, studying the influence of olive ripening on the fatty alcohol composition analyzed by gas chromatography [35] and in food analytical methods, using liquid chromatography and mass spectrometry to analyze polyphenols [33]. This last article was also the one with the fewest citations (only 13 by the end of 2018 for an IF of 2.413).

### 3.3. Main Authors

A total of 2168 authors have contributed to at least one of the 732 studied articles. However, the vast majority (73%) do not show a strong interest in the subject of olive oil origin since they only appear in a single article between 1991 and 2018. Only 22 authors have published 10 articles or more in this period. Table 3 shows the evolution of the number of publications for these most productive authors. Some of them have taken an early interest in the subject: Reniero published an article in 1993, followed by Forina in 1994. On the contrary, Bajoub has demonstrated the most recent but quite strong interest with a contribution to 14 articles since 2014. Most of these main authors appear to still be active with at least one publication since 2015, except for Cerretani and Downey whose activity was restricted to the period between 2003 and 2014. Looking at the most cited articles from each author indicates that some of them have worked in collaboration with each other (Table 3) [17,18,19,20,36,37,38,39,40,41,42,43,44,45,46]. This was the case for Artaud and Dupuy [19] using near-infrared spectroscopy and chemometrics, Casale and Forina [20] applying chemometrics to combine data from e-nose, UV-Visible and near-infrared spectroscopy, Fernandez-Gutierrez and Zarrouk [36] as well as Bajoub and Carrasco-Pancorbo [40] analyzing phenolic compounds by liquid chromatography and mass spectrometry, Bendini and Cerretani [39] predicting sensory attributes with chemometric models using near- and mid-infrared spectroscopy and finally Del Coco and Fanizzi [42] or Guillou and Reniero [43] applying chemometrics to nuclear magnetic resonance data.

### 3.4. Main Countries

**Figure 2:**
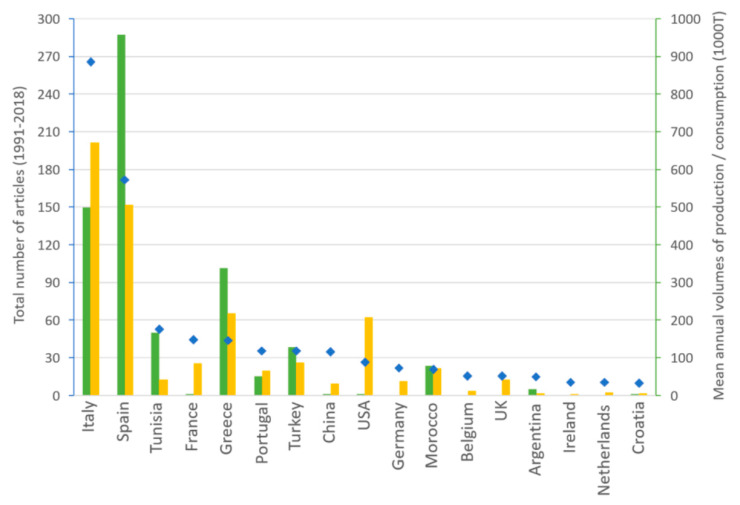
Annual volumes of olive oil production (green bars) and consumption (yellow bars) for the countries having published more than 10 articles (blue diamonds) between 1991 and 2018.

The number of articles published by each country can be compared to the volumes of olive oil production and consumption obtained from the International Olive Oil Council report [47]. Figure 2 presents the annual volumes of olive oil production and consumption for the countries having published more than 10 articles between 1991 and 2018. It indicates that Italy and Spain were by far the most productive countries, both in terms of articles and volume of olive oil produced. However, there was a stronger scientific interest for olive oil origin in Italy, which may be related the higher number of oils with a certification of origin: 42 olive oils with a PDO and 4 with a PGI were produced in Italy versus 29 with a PDO in Spain [48] and also to the very high number of cultivars: 538 cultivars were listed in Italy versus 262 in Spain [49]. The interest of Tunisia, Greece, Portugal, Turkey and Morocco in studying olive oil origin seems consistent with their position as olive oil producing countries, even though Greece has fewer articles and Portugal more articles than expected from their respective production volumes and numbers of PDO (19 in Greece and 6 in Portugal), PGI (11 in Greece and none in Portugal) [48] and cultivars (52 in Greece and 24 in Portugal) [49]. The number of articles from France, the USA and to a lesser extent Germany and the UK can be explained by their relatively high consumption of olive oil and for France by the existence of 8 PDO olive oils, despite a low production. However, other countries such as Belgium, Argentina, Ireland, The Netherlands, Croatia—and more importantly, China—have a higher number of publications than would be expected from their volumes of olive oil production or consumption.

### 3.5. Data Clustering

**Figure 3:**
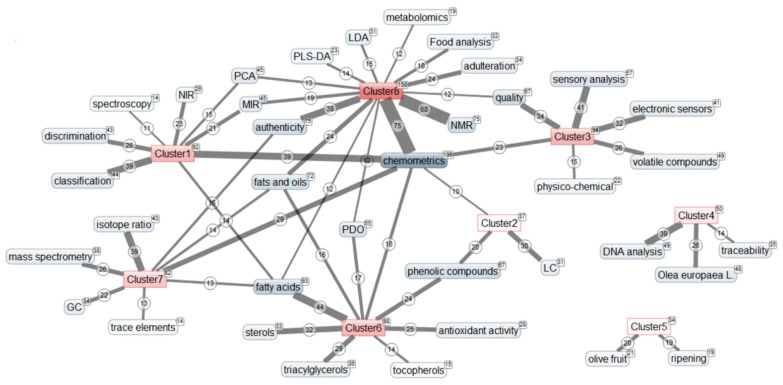
Network of the main keywords associated at least ten times with one or more of the thematic clusters (□: total number of articles in the corpus associated with this keyword or cluster, ○: number of articles associated with this keyword in the specified cluster). GC: gas chromatography; LC: liquid chromatography; MIR: mid-infrared; NIR: Near-infrared; NMR: Nuclear Magnetic Resonance; PDO: protected designation of origin; PCA: principal component analysis; LDA: linear discriminant analysis; PLS-DA: partial least square-discriminant analysis.

**Figure 4:**
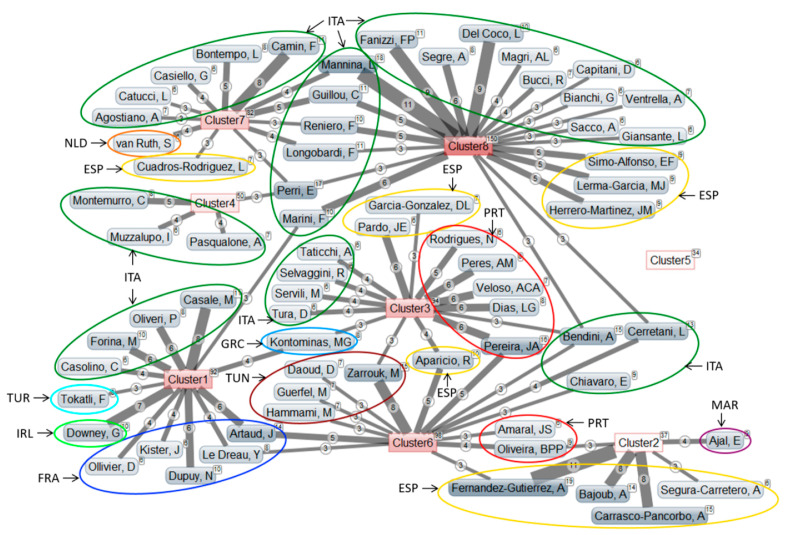
Network of the main authors, having published at least 6 articles and associated at least 3 times with one or more of the thematic clusters, with their country (ESP: Spain, FRA: France, GRC: Greece, IRL: Ireland, ITA: Italy, MAR: Morocco, NLD: The Netherlands, PRT: Portugal, TUN: Tunisia, TUR: Turkey, □: total number of articles in the corpus associated with this author or cluster, ○: number of articles associated with this author in the specified cluster).

In order to reveal the existence of some thematic groups which structure this research, keywords that were present in at least ten articles, excluding the words used in the search query (i.e., “olive oil”, “geographical origin” and “cultivar”), were subjected to a K-means analysis [50] in Matheo Analyzer^®^ resulting in their partition into eight clusters. Figure 3 shows the resulting network of keywords and their distribution into the eight thematic clusters. To keep it easily readable, only the keywords associated at least ten times with a cluster were represented. Table 4 presents the evolution of the number of publications in the clusters between 1991 and 2018 and Figure 4 shows the network of the main authors (with the indication of their nationality), having published at least 6 articles and associated at least 3 times with one or more of the thematic clusters. The results of this analysis should be considered with caution since they depend on the database from which the articles were retrieved (in this case: Web of Science) and on the keywords used in each article, stressing once more the importance for authors to carefully choose the most suitable keywords in their articles.

Some keywords were present in several clusters (Figure 3). For instance, “chemometrics” was present in all but two clusters, although it was mainly related to cluster 8. Similarly, “fatty acids” was mostly found in cluster 6 and to a lesser extent in clusters 1, 7 and 8, while “fats and oils” was divided between clusters 6, 7 and 8. However, since most of the keywords can be mainly attributed to one specific cluster, the theme of each cluster can be identified. The network of relationships between thematic clusters and the main authors (Figure 4) indicates the orientation of their research. Most authors were associated with a single topic, although some of them appear to create bridges between two or three thematic clusters. As could be expected, authors from the two most productive countries, Italy and Spain, were present in a wide range of themes.

#### 3.5.1. Cluster 1

This cluster was focused on near- and mid-infrared analytical techniques, combined with chemometrics classification and discrimination models (Figure 3). It has been studied consistently throughout the years but has known a decline of popularity since 2015 (Table 4). Authors from several countries take an interest in this subject, including Casale and others in Italy, Artaud and colleagues in France, Downey in Ireland, Tokatli in Turkey and Kontominas in Greece (Figure 4). This cluster was characterized by an intense use of chemometric methods, which justifies the strong presence of specialists in this field like Downey, Dupuy and Marini.

#### 3.5.2. Cluster 2

This small group of articles deals with the application of liquid chromatography to analyze phenolic compounds (Figure 3). Interest in this subject was more recent, with a first article in 2002, and has seen a strong increase in the 2015–2018 period (Table 4). This field benefits from technological advances with instrumentation allowing the analysis of compounds present at very low concentrations. It was mostly studied by Spanish researchers such as Fernandez-Gutierrez and others, but also by Ajal in Morocco and Oliveira in Portugal (Figure 4).

#### 3.5.3. Cluster 3

Articles of the cluster 3 were centered on sensory analysis and the use of electronic sensors, related to the analysis of volatile compounds. They also study the quality and physicochemical parameters of olive oil (Figure 3). These themes have been increasingly studied between 1991 and 2014, but the number of articles has been stagnating since 2015, with only 28 and 29 articles for the two most recent periods (Table 4). The subject attracts researchers from various countries like Pereira and others in Portugal, Pardo and Garcia-Gonzalez in Spain, Tura and colleagues in Italy, Kontominas in Greece and Zarrouk in Tunisia (Figure 4). This cluster was strongly connected to chemometrics methods, like the cluster 1.

#### 3.5.4. Cluster 4

This isolated group covers the use of DNA analysis to insure the genetic traceability of *Olea europaea subsp europaea* L. (Figure 3). This rather recent subject was increasing studied since the first article published in 2000 (Table 4). This type of analysis concerns only a few authors and remains the responsibility of specialists. It was specifically studied by Italian researchers, with Montemurro and colleagues (Figure 4).

#### 3.5.5. Cluster 5

This small and isolated cluster containing only 34 articles seems to be somewhat outside of the main thematic at first sight, since it concerns olive fruit and ripening (Figure 3). However, a closer look indicates that these articles actually deal with the influence of olives maturity degree on the composition and quality of the resulting olive oil. It was the opening door to a much broader work that relates to the nutritional impact of olive oils in relation to their chemical composition and more particularly with phenolic compounds, antioxidants and vitamins. Interest in this subject has been growing sharply in recent years (Table 4), even though none of the main authors appears to be strongly connected to this subject so far due to its novelty (Figure 4).

#### 3.5.6. Cluster 6

This theme gathers the analysis of various compounds such as fatty acids, triacylglycerols, sterols, tocopherols, phenolic compounds and the study of antioxidant activity (Figure 3). It has been studied since 1991 but with a strong increase of publications between 2003 and 2010 and stagnation since 2011 (Table 4). It was mostly studied by researchers that were also interested in another subject, including Zarrouk and others in Tunisia, Artaud and Le Dréau in France, Aparicio and Fernandez-Gutierrez in Spain, Pereira, Oliveira and Amaral in Portugal, and Cerretani, Bendini and Chiavaro in Italy (Figure 4).

#### 3.5.7. Cluster 7

This cluster was mainly related to isotope ratio, mass spectrometry and gas chromatography to analyze trace elements, as well as fatty acids (Figure 3). It has known a steady increase of popularity since 1998 and has become the second most studied theme in the recent years (Table 4). It attracts a large number of Italian researchers, like Camin and colleagues, but also Cuadros-Rodriguez in Spain and van Ruth in The Netherlands (Figure 4).

#### 3.5.8. Cluster 8

The 150 articles that compose this largest cluster were part of the “omics” movement (metabolomics), with the processing of infrared and nuclear magnetic resonance data by chemometrics methods such as partial least square discriminant analysis, principal component analysis or linear discriminant analysis in order to solve problems of adulteration and determination of quality parameters of olive oils (Figure 3). It was the most popular theme, with an especially strong increase of publications since 2007 (Table 4). Once again, this subject was largely studied by Italian researchers who focus specifically on this area, like Fanizzi and colleagues, or have interest in other clusters like Mannina and others who were also connected to gas chromatography or isotopic ratio analyses (cluster 7), Marini who was also involved in vibrational spectroscopic analyses (cluster 1), or Bendini and others who were also concerned with sensory analyses and quality (cluster 3) and with target compounds (cluster 6). A team of Spanish researchers with Simo-Alfonso, Lerma-Garcia and Herrero-Martinez was also connected to cluster 8 (Figure 4).

## 4. Conclusions

It is clear that the scientific interest in olive oil origin has been consistently increasing since the early 1990s, concurrently with the growing consumption of this product and awareness of authenticity issues. Indeed, olive oil is one of the most extensively studied edible oils and has served as a reference to develop the concepts of varietal and geographical origin discrimination. This bibliometric study highlights the core journals in which research articles on this topic are most likely to be published, the most prominent authors with their specific areas of expertise and the relationships between the scientific and economic interests of the most productive countries. The 732 references published between 1991 and 2018 can be distributed into eight clusters by a K-means analysis performed on their keywords, allowing to identify the main themes of research. A shift of popularity seems to be occurring from chemical fingerprinting using vibrational spectroscopy towards biologic phenotyping using genetic and metabolomic techniques, as indicated by the evolution of the number of publications in the corresponding clusters. Chemometric tools are now well established and are expected to continue to be increasing applied to treat the results from various analytical techniques. Moreover, the presence of connections creating a large network between most of the thematic clusters indicates the potential for multimethodological studies combining for instance infrared spectroscopy with gas chromatography or nuclear magnetic resonance with isotopic ratio or with sensory analysis. Finally, a trend to focus on the sensory and nutritional properties brought by minor compounds of olive oils appears to be emerging. Quantifying the minor compounds of olive oil leads to further study the complex relationships between the varietal origin, the ripening stage of the olives and the nutritional quality of the oil.

## Figures and Tables

**Table 1:** Number of articles containing keywords related to specific analytical techniques and most cited article for each technique, with citation count up to 2018.

Analytical Technique	Articles	Most Cited Article	Citation Count
Nuclear magnetic resonance	75	[11]	142
Sensory analysis	57	[12]	189
DNA analysis	49	[13]	83
Electronic sensors	41	[14]	301
Isotope ratio	40	[15]	96
Mid-infrared spectroscopy	40	[16]	181
Gas chromatography	34	[17]	141
Liquid chromatography	31	[18]	201
Near-infrared spectroscopy	28	[19]	129
UV-Visible spectroscopy	16	[20]	80
Raman spectroscopy	9	[21]	117
Fluorescence spectroscopy	7	[22]	84

**Table 2:** Journals having published at least 10 articles between 1991 and 2018, sorted by their respective number of articles, with their associated impact factor (IF), subject category(ies), ranking in each category notified by their quartile (Q), most cited article and citation count up to 2018.

Journal	Articles	IF 2018	Category: Q 2018	Most Cited Article	Citation Count
Food Chemistry	94	5.399	Chemistry (Applied): Q1, Food Science: Q1, Nutrition: Q1	[23]	269
Journal of Agricultural and Food Chemistry	66	3.571	Agriculture: Q1, Chemistry (Applied): Q1, Food Science: Q1	[24]	405
Journal of the American Oil Chemists Society	43	1.720	Chemistry (Applied): Q2, Food Science: Q2	[12]	183
European Journal of Lipid Science and Technology	38	1.852	Food Science: Q2, Nutrition: Q3	[25]	40
Food Research International	25	3.579	Food Science: Q1	[26]	95
European Food Research and Technology	22	2.056	Food Science: Q2	[27]	70
Journal of the Science of Food and Agriculture	21	2.422	Agriculture: Q1, Chemistry (Applied): Q2, Food Science: Q2	[28]	51
Analytica Chimica Acta	20	5.256	Chemistry (Analytical): Q1	[14]	301
Grasas y Aceites	18	0.891	Chemistry (Applied): Q4, Food Science: Q4	[29]	46
Talanta	15	4.916	Chemistry (Analytical): Q1	[30]	61
Rivista Italiana delle Sostanze Grasse	14	0.694	Food Science: Q4	[31]	15
Journal of Chromatography A	10	3.858	Biochemical Research Methods: Q1, Chemistry (Analytical): Q1	[32]	108
Food Analytical Methods	10	2.413	Food Science: Q2	[33]	13
Critical Reviews in Food Science and Nutrition	10	6.704	Food Science: Q1, Nutrition: Q1	[34]	113
Journal of Oleo Science	10	1.208	Chemistry (Applied): Q3, Food Science: Q3	[35]	23

Q1: first quartile (rank in top 25%), Q2: second quartile (rank between 25% and 50%), Q3: third quartile (rank between 50% and 75%), Q4: fourth quartile (rank over 75%).

**Table 3:** Number of publications over time for the main authors (having a total of at least 10 articles) and most cited article for each author with its citation count up to 2018.

	1991–1994	1995–1998	1999–2002	2003–2006	2007–2010	2011–2014	2015–2018	Total	Most Cited Article	Citation Count
Fernandez-Gutierrez, A	-	-	-	-	2	4	13	19	[36]	64
Mannina, L	-	1	3	3	5	1	5	18	[37]	125
Perri, E	-	-	3	-	5	6	3	17	[38]	112
Pereira, JA	-	-	1	2	3	2	8	16	[18]	201
Bendini, A	-	-	-	-	7	4	4	15	[39]	90
Carrasco-Pancorbo, A	-	-	-	-	1	1	13	15	[40]	21
Zarrouk, M	-	-	-	1	5	5	4	15	[36]	64
Artaud, J	-	-	-	3	5	2	4	14	[19]	129
Bajoub, A	-	-	-	-	-	1	13	14	[40]	21
Cerretani, L	-	-	-	-	8	5	-	13	[39]	90
Camin, F	-	-	-	-	3	1	7	11	[41]	80
Casale, M	-	-	-	1	7	1	2	11	[20]	80
Fanizzi, FP	-	-	-	-	-	4	7	11	[42]	24
Guillou, C	-	-	4	1	4	1	1	11	[43]	119
Longobardi, F	-	-	-	-	-	6	5	11	[44]	70
Aparicio, R	-	-	-	5	2	1	2	10	[17]	141
Del Coco, L	-	-	-	-	-	4	6	10	[42]	24
Downey, G	-	-	-	1	7	2	-	10	[45]	169
Dupuy, N	-	-	-	1	5	2	2	10	[19]	129
Forina, M	1	-	-	1	6	1	1	10	[20]	80
Marini, F	-	-	1	2	3	3	1	10	[46]	98
Reniero, F	1	-	5	1	2	-	1	10	[43]	119

**Table 4:** Evolution of the number of publications in the thematic clusters between 1991 and 2018.

	1991–1994	1995–1998	1999–2002	2003–2006	2007–2010	2011–2014	2015–2018	Total
Cluster 1	2	1	10	8	22	31	18	92
Cluster 2	-	-	1	4	5	7	20	37
Cluster 3	2	1	3	12	19	28	29	94
Cluster 4	-	-	4	5	9	11	21	50
Cluster 5	1	1	2	2	7	5	16	34
Cluster 6	1	1	2	13	26	27	28	98
Cluster 7	-	2	2	5	13	22	38	82
Cluster 8	-	5	11	10	24	44	56	150
